# The Role of NLRP3 Inflammasome Activation in the Epithelial to Mesenchymal Transition Process During the Fibrosis

**DOI:** 10.3389/fimmu.2020.00883

**Published:** 2020-05-20

**Authors:** Amani Abraheem Alsadiq Alyaseer, Murilo Henrique Saturnino de Lima, Tarcio Teodoro Braga

**Affiliations:** ^1^Department of Pathology, Federal University of Parana, Curitiba, Brazil; ^2^Instituto Carlos Chagas, Fiocruz-Parana, Curitiba, Brazil

**Keywords:** NLRP3, EMT—epithelial to mesenchymal transition, fibrosis, inflammasome, TGF-β

## Abstract

Fibrosis is considered a complex form of tissue damage commonly present in the end stage of many diseases. It is also related to a high percentage of death, whose predominant characteristics are an excessive and abnormal deposition of fibroblasts and myofibroblasts -derived extracellular matrix (ECM) components. Epithelial-to-mesenchymal transition (EMT), a process in which epithelial cells gradually change to mesenchymal ones, is a major contributor in the pathogenesis of fibrosis. The key mediator of EMT is a multifunctional cytokine called transforming growth factor-β (TGF-β) that acts as the main inducer of the ECM assembly and remodeling through the phosphorylation of Smad2/3, which ultimately forms a complex with Smad4 and translocates into the nucleus. On the other hand, the bone morphogenic protein-7 (BMP-7), a member of the TGF family, reverses EMT by directly counteracting TGF-β induced Smad-dependent cell signaling. NLRP3 (NACHT, LRR, and PYD domains-containing protein 3), in turn, acts as cytosolic sensors of microbial and self-derived molecules and forms an immune complex called inflammasome in the context of inflammatory commitments. NLRP3 inflammasome assembly is triggered by extracellular ATP, reactive oxygen species (ROS), potassium efflux, calcium misbalance, and lysosome disruption. Due to its involvement in multiple diseases, NLRP3 has become one of the most studied pattern-recognition receptors (PRRs). Nevertheless, the role of NLRP3 in fibrosis development has not been completely elucidated. In this review, we described the relation of the previously mentioned fibrosis pathway with the NLRP3 inflammasome complex formation, especially EMT-related pathways. For now, it is suggested that the EMT happens independently from the oligomerization of the whole inflammasome complex, requiring just the presence of the NLRP3 receptor and the ASC protein to trigger the EMT events, and we will present different pieces of research that give controversial point of views.

## Fibrosis

The inflammatory response is triggered in an attempt to remove an aggressor agent and ultimately leads to the wound healing process as a result of reparative or reactive steps in different organs concomitant with the elimination of the agent. This agent can be a pathogen, in which the inflammatory response is initiated after the recognition of a PAMP (Pathogen Associated Molecular Pattern), or the agent can be an endogenous product, named DAMP (Damage Associated Molecular Pattern), both recognized by PRRs (Pattern Recognition Receptors), which are receptors of innate immune system (1). Although it is considered a normal process for maintaining the architecture and functional integrity of the affected organ, if the injurious stimuli persist, such a response will result in the deregulation of normal processes and scar formation.

Fibrosis can be defined as an accumulation of fibrous connective tissue, in particular extracellular matrix (ECM) components such as type I collagen and fibronectin (2), the accumulation of which can cause the malfunction of the organ and its failure. This process is the end result of chronic inflammatory diseases such as rheumatoid arthritis (3), idiopathic pulmonary fibrosis (4, 5), Chron's Disease (5), and Chronic Kidney Disease (CKD) (6, 7) besides autoimmune reactions, allergic responses, chemical insults, radiation, and tissue injury (6), all of which progress to an advanced state characterized by a poor quality of life of the patient, mainly due to the lack of specific treatments for the reversibility of the fibrotic process (2). The origin of the fibrosis process is, therefore, an unresolved inflammatory response (8) or the improper removal of stimulus, which culminates in a chronic inflammatory response (9). The strategies to deal with fibrotic diseases rely on an attempt to neutralize the aggressor but not all of them are easily diffusible or with a known origin, which forces clinicians to try to find ways to control and reverse the fibrosis (2). Inflammatory mediators are responsible for controlling the response triggered against aggressor agents and for starting the tissue repair process. In chronic inflammatory diseases, macrophages are the main characters in expressing these mediators (10). These cells are differentiated, mainly, from monocytes when they leave the circulatory system and invade the tissue to become phagocytes and are able to produce inflammatory cytokines (11). Besides bone marrow hematopoietic cells, macrophages are also derived from different sources, including local proliferation of embryonic progenitor cells, which is responsible for the generation of tissue-resident macrophages (12). They are plastic cells and functionally subdivided into profiles that vary from an inflammatory subpopulation (usually called M1 macrophages) to an anti-inflammatory one (usually referred as M2 macrophages) (13). It is noteworthy to state that anti-inflammatory macrophages can be derived from inflammatory ones or resident cells, in contrast to inflammatory cells which are derived mainly from circulating monocytes. Additionally, inflammatory macrophages express IL-1β, IL-23, TNF-α, and lipid-derived mediators (14, 15). Another important characteristic of the inflammatory profile is the production of reactive oxygen species (ROS), which is necessary to kill the invader and therefore responsible for the injury during inflammation (14, 15). The anti-inflammatory cells, on the other hand, appear to exert the opposite effect, i.e., controlling the inflammatory process. This subpopulation is responsible for producing IL-10, IL-4, and transformation growth factor-β (TGF-β) (15), playing, therefore, a role in the clearance of apoptotic cells and the formation of wound healing, mainly due to the expression of TGF-β, although it has not been shown that the presence of anti-inflammatory macrophages in the absence of an inflammatory context leads to the formation of fibrotic tissue.

### Epithelial to Mesenchymal Transition

Epithelial to mesenchymal transition (EMT) is a biological process, found in the context of fibrosis, that is characterized by the changing in the phenotype of polarized epithelial cells into mobile mesenchymal cells through a switch of the expressed proteins (16). During EMT it has been observed that there is an up-regulation of α-sma (α-smooth muscle actin), a marker of mesenchymal cells, and a downregulation of e-cadherin, the key marker of epithelial cells responsible for the lateral adherent junctions and adhesion (17). Usually, epithelial cells have a square-shaped morphology and they are strongly adherent to their basement membrane and the neighbor cells, ultimately being responsible for maintaining the integrity of cell junctions (18). However, after a process of EMT, induced by a constellation of signaling pathways, whether intrinsic (such as genetic mutations) or extrinsic (such as growth factors including TGF-β, the WNT, and Sonic Hedgehog), those cells will gradually start losing their ability to express their known adhesion proteins, structure, and function to end up turning to the mesenchymal cells. The EMT process converges in an increase of transcription factors such as snail, ERK, and slug which initiate and maintain the process of EMT (19). The outcome of the EMT process is the changing morphology of epithelial cells into spindle-shaped form cells that do not adhere to other cells, but instead present an enhanced migratory capacity, the ability of invasion, and a high resistance to apoptosis (18). The EMT process does not represent a bipolarity between epithelial and mesenchymal cells, but instead is a spectrum of different protein expression throughout the time that culminates in a complete transition. Both EMT and the opposite process, known as mesenchymal to epithelial transition (MET), are considered integral stages of many physiological processes that are tightly coordinated by several molecular regulators (20–22). The down-regulation of the epithelial markers that have been observed in the EMT process is also mediated by mesenchymal markers up-regulation such as vimentin, n-cadherin, integrin, fibronectin, and matrix metalloproteinase, which will result in loss of the cytoskeleton polarization and development of lamellipodia and, thus, an increase in its cell motility. Additionally, the EMT process is also responsible for the increase in extracellular matrix deposition (18, 23).

EMT plays a main role in diverse biological processes other than the fibrotic process, such as embryogenesis and cancer metastasis. It is separated into three types, each of them presenting a different function in the mesenchymal cells at the end of the process (16, 18, 24). The type I EMT is present in embryogenesis implantation, during the initiation of the placenta development and organ development; the type II EMT, on the other hand, occurs during fibrotic diseases in organs such as the liver, kidney, lung, and intestine, and it is also associated with tissue regeneration and wound healing; the type III EMT is conceptually present in the progression of cancer and metastasis (18, 25). In the early days of a mammal's embryo development, a structure called blastocyst, composed of a blastocoele and a trophoblast, triggers type I EMT to invade and adhere to the endometrium to subsequently form the placenta (26). In cancer, the cells can migrate to another tissue via vessels. This ability is attributed to the type III EMT that promotes a loss of cell adhesion and helps the posterior invasion. Both type I and type III EMT processes are reversible by the MET process after a tissue invasion, a reversion that does not happen in type II EMT (27). During fibrotic diseases, the main triggered mechanism is the type II EMT. Here, the mesenchymal type of cells formed after complete transitions are myofibroblasts and fibroblasts, responsible for expressing ECM components in tissue repair after an injury (16). In the present review, we will give special attention to type II EMT due to its importance in fibrosis.

#### Type II EMT

In inflammation, after damage is caused by infectious agents, toxins, allergens, or trauma, a release of DAMPs, mainly due to cellular death, which are latter recognized by PRRs such as like Toll-Like receptors (TLR) and Nod-like receptors (NLR), become the major element of the inflammasome complex (28). This recognition promotes the assembly of an inflammatory response that aims to destroy the aggressor agent in order to control and repair the tissue damage. To repair this damaged tissue, myofibroblasts are recruited to the lesion site and start to release metalloproteinases and digestive enzymes. After the clearance, myofibroblasts produce new ECM proteins to promote the wound healing (29, 30). The origin of myofibroblasts is due to a high expression of TGF-β, responsible for triggering EMT in epithelial cells through its receptor (31). Any small flaw in this mechanism might result in the promotion of a large amount of ECM components synthesis, leading to an exacerbated scar formation and compromising the tissue function (29, 30).

The intracellular signaling pathways involved in EMT are complex and can cross-talk between them. TGF-β was first described as an inducer of EMT in normal mammary epithelial cells by signals through the serine/threonine kinase complexes receptor (32). The signaling pathway better described is the TGF-β/Smad pathway. As EMT represents a gradual transition from an epithelial cell type to a mesenchymal one, the first signals are responsible for forming a hybrid cell type that is partially epithelial but already starts the expression of mesenchymal markers, a process mediated by ERK, snail, and slug (19). This process can be reversible once the damage stimuli are removed and the hybrid return to an epithelial form (19). The TGF-β pathway is a complex signaling mechanism involving different proteins, and, additionally, may be a Smad-independent pathway (19), but Rhoa-dependent, a GTPase-related enzyme. This enzyme is related to the cytoskeletal modeling as it activates the phosphorylation of the downstream ROCK I/II, a serine/threonine kinase-specific protein that acts in actin protein (33, 34). Another important signal is MAPK/ERK. Once TGF-β binds into its receptor, it activates an upstream GTPase RAS responsible for triggering a set of mitogen-protein kinases, the last of which is the MAPK/ERK (35). This pathway was shown to establish a relationship with EMT once an inhibition of this signal interrupts EMT (34). Phosphatidylinositol 3-Kinase (PI3K) is also a signal activated by TGF-β (36) and it has been linked to EMT as its inhibition also stops the transition processes in the HK-2 cell model, a renal epithelial cell line (37). But the exact mechanism remains unknown in tubulointerstitial fibrosis (34).

*In vitro* models of EMT have been used in the past few years to elucidate the mechanism and therapeutics targets of EMT in several diseases that compromise vital organs like the lungs, heart, and kidneys. In pulmonary fibrosis, for instance, the induction of EMT can be done by different inducers such as bleomycin, silica, and the atmospheric particular matter (38–40). All of them are responsible for triggering EMT as observed by the increase of mesenchymal markers such as FSP-1 and α-sma and a decrease of e-cadherin. In other tissues, other inducers are shown to be successful in modulating the EMT markers as TGF-β in kidney diseases (41–44) and heart diseases (45). In the next sections, we will give special attention to some EMT modulators.

### TGF-β

Transforming growth factor-β (TGF-β) is a cytokine member of a large family which carries the same name and is linked with several essential cellular functions, including embryological development, fibrosis, and cancer (46). Early research studying cancer biology found out the mechanisms by which TGF-β acts (47) and its name was termed due to the incorrect belief that this cytokine was only expressed in transformed cells during the establishment of cancer (47). Other members that belong to this family are bone morphogenetic protein-7 (BMP-7), activin, nodal, and anti-mullerian hormone, which are all structurally related molecules and are all involved in critical cellular functions such as proliferation, differentiation, motility, extracellular matrix production, angiogenesis, and apoptosis (48, 49).

In addition to the known anti-inflammatory effect of TGF-β, it has also shown to have the ability to act as a profibrotic agent, through the increase of ECM synthesis and the expression of proteinase inhibitors, including plasminogen activator inhibitor-1 (PAI-1), and by decreasing the ECM-degrading proteins, such as collagenase (50). Moreover, according to both Li et al. and Strutz et al. this profibrotic activity is a potent inducer of EMT and part of the TGF-β-induced EMT program which promotes the remodeling of cell contacts with the basal membrane (51, 52). Besides, such cellular contact remodeling is triggered by the activation of the matrix metalloproteases MMP2 and MMP9 which also results in the degradation of type IV collagen, a component of the basement membrane (51, 52). In humans, TGF-β exists in three main isoforms (TGF-β1, TGF-β2, and TGF-β3) with TGF-β1 being the most common (53) and, although they are encoded by distinct genes, they share a similar homology and activate the same intracellular signaling pathway (54). TGF-β1 is also responsible for the formation of myofibroblasts through the activation of resident fibroblasts and induction of both EMT and endothelial to mesenchymal transition (EndMT) (55, 56). Mice overexpressing an active form of the TGF-β1 in the liver, for instance, have shown to develop progressive liver and renal fibrosis (57, 58). Other experimental models using knockout mice led to a lethal multifocal inflammatory disease, which shows that they also play different roles in maintaining homeostasis and in embryogenic development (59–61). Moreover, the blockage of TGF-β 1 signaling either in T cells or in bone marrow results in a similar multifocal inflammatory responses (62, 63). The three isoforms of TGF-β are also secreted as inactive latent precursors that require activation before binding to the TGF-β receptors (64).

All isoforms of TGF-β interact with two receptors in the cell membrane, the type I receptor (TGF-βRI) and the type II receptor (TGF-βRII), which are both threonine/serine kinase receptors. When TGF-β link to TGF-βRI, this promotes the phosphorylation of TGF-βRII, which will posteriorly recruit downstream signals in a Smad-dependent pathway (65), a signaling pathway better explored in the next section. The complex Smad2 and Smad3 form a trimer with the common Smad4 to reach the nucleus and start the transcription of genes related to different cellular functions (65). Additionally, TGF-β can activate a number of non-Smad signaling pathways where the activated receptors also signal through other transducers, such as the mitogen-activated protein kinase (MAPK) pathways, that includes each of the extracellular signal-regulated kinases (ERKs), AKT, Rho family GTPases, c-Jun amino-terminal kinase (JNK), and p38 MAPK, as well as the IkB kinase (IKK) and phosphatidylinositol-3 kinase (PI3K) (66, 67), as stated above. These non-Smad signals play different roles: they are involved in TGF-β-mediated biological responses, but can also regulate the canonical Smad pathway (68).

### Smad

Smad, named due to the members Mad (the Drosophila gene Mothers Against Decapentaplegic) and Sma (the Mad homologs observed in the nematode *Caenorhabditis elegans*) (69) have shown to be activated by numerous upstream molecules with pleiotropic functions and are thought to constitute the core-point for transcriptional regulation of multiple genes with diverse activities (70). Smad are proteins with a transcription factor function, responding to TGF-β signaling after phosphorylation of their receptors' activities (70). They are categorized into two subgroups according to their functions, i.e., R-Smad, the receptor-activated Smad, and I-Smad, the inhibitory Smad. R-smad are phosphorylated by either TGF-βRII (include Smad-2 and Smad-3) or by BMP receptor (BMPR) (and include Smad1, Smad-5, and Smad-8). These R-Smad bind to the Smad-4, also known as the Co-Smad, which form a heterodimer with the activated phosphorylated R-Smad, and, together, move from the cytoplasm to the nucleus, initiating the transcriptional activity (71). I-Smad (which includes Smad-6 and Smad-7), on the other hand, inhibit the signaling activity of the R-Smads and the formation of the Co-Smad/R-Smad complex, important for creating a balance during non-fibrotic functions (70, 72).

It has been described that both Smad2 and Smad3 promote EMT in the context of fibrosis after TGF-β signaling pathway activation. Moreover, based on knockout studies, the specific role of Smad2 or Smad3 has been investigated (73). TGF-β1-mediated induction of MMP-2 was selectively dependent on Smad2, whereas activation of the activin response was strongly suppressed in Smad2 KO fibroblasts but enhanced in Smad3 KO cells (73). The Smad proteins consist of a highly conserved amino-terminal MH1 domain and a carboxyl-terminal MH2 domain, which are separated by a proline-rich linker region (74). Both Smad2 MH1 and MH2 domains' knockout approaches led to lethality in early stages of mice embryonic development, due to a misormation of an anterior-posterior axis, gastrulation, and mesoderm (75, 76), suggesting that Smad2 is more closely related to embryogenic events than others following the TGF-β/Smad pathway. Smad3 KO mice showed viability in newborn mice and guaranteed survival for a few months but led to systemic chronic inflammation and death when left for a longer time (73). Therefore, Smad3 is not important to embryological development but it is crucial in the pathobiology of fibrosis development (77). Although both Smad2 and Smad3 are strongly activated in liver fibrosis (78), Smad3 appears to be the key element in the signal transduction pathways that are responsible for fibrosis (79). Smad3 activation leads to an increased expression of fibrogenic genes, including α-sma and e-cadherin (80, 81). Besides, Smad3 activation through TGF-β signaling triggers the induction of tissue inhibitor of metalloproteinases (TIMP) (82) and thus prevents the degradation of ECM (77). Therefore, Smad3 knockdown results in the blocking of EMT and attenuation of renal fibrosis, inflammation, and apoptosis in a model of unilateral ureteral obstruction (UUO) (83).

Additionally, some inactivating mutation into Smad genes were described in the cancer context. DPC4 (deleted in pancreatic carcinoma locus 4), for instance, was identified as a tumor suppressor gene in carcinoma, implicated in Smad4 signaling (84). Early colorectal carcinoma was found to be associated with Smad2 and Smad4 inactivating mutations (85, 86). Despite some reports pointing to an absence of acquired mutations, insertions, or microdeletions in Smad3 in the skin and parathyroid tumors (87), its somatic mutation associated with Smad2 and Smad4 mutations is a cause of sporadic colorectal carcinoma (88). Smad3 also promotes cancer progression by inhibiting E4BP4-mediated NK cell development (89). Accordingly, Smad3 functions as both a negative and positive regulator of carcinogenesis depending on cell type and clinical stage of the tumor (90).

### BMP-7

Bone morphogenic protein 7 (Bmp-7), also known as osteogenic protein 1, is a member of the transforming growth factor-β proteins superfamily (48, 49). The Bmp-7 compromises an anti-inflammatory, anti-fibrotic, anti-apoptotic, and a proliferating stimulating function (91–93). It also exerts a crucial role in kidney development, whereas Bmp-7 KO mice die after birth due to renal failure (94, 95). Like TGF-β, Bmp-7 signaling is also mediated by the heterodimeric receptors with serine/threonine receptor kinases and the cytoplasmic proteins Smads (96), but while the TGF-β acts on the TGF-βR to phosphorylate certain types of Smad, the Bmp-7 binds to the BMPIIRs to activate R-Smad1/5/8 (96, 97) and the activin-like kinase (ALK) receptors ALK2, ALK6, and ALK3. Moreover, BMP-7 can signal through non-canonical pathways to exert its biological activity (65, 98). Although the precise function of Bmp-7 is not well-understood, researches have proven that Bmp-7 acts as an endogenous regulator of organ homeostasis and regeneration in the kidney and liver (91, 99). Hence, recombinant Bmp-7 reduces the severity of injury in acute and chronic organ failure by counteracting profibrogenic TGF-β activities (100).

Studies have also shown that Bmp-7 is effective in reversing both EMT and the fibrotic effect that is induced by TGF-β; this was proved by Ziesberg's lab team in 2003, who focused on chronic renal injury, and by Ying and colleagues in 2017, where they studied the inhibition of TGF-β -induced EMT using BMP-7 in breast cancer (93, 101). In other words, Bmp-7 acts as an opposing factor to TGF-β, thereby reducing inflammation and ECM-related products, such as type I and type III collagen, that are hallmarks of organ fibrosis (102). Moreover, Higgings and colleagues proved in an experimental study both *in vivo* and *in vitro* that this inhibition is done through the decreased activation of PI3K signaling via Akt and also the inhibition of Smad3 phosphorylation without any effect reported on Smad2 or the Smad2/3/4 complex (103). Another study by Luo et al. showed that this inhibition was caused by interference with Smad3 DNA binding site and the enhancement of SnoN expression (104). Other studies that were concerned with the function of BMP-7 showed that it played a role in preserving the blood flow, preventing tubular atrophy, and reducing tubular interstitial fibrosis in obstructive neuropathy in rats (105). Additionally, in an acute tubular necrosis model performed by Vukicevic's team, it was proved that BMP-7 preserves the kidney function and increases the patient's survival rate (100). Zeisberg et al. using mice with acute glomerular nephritis, showed that BMP-7 induces EMT reversion by increasing the expression of e-cadherin and leading to ECM degradation (93).

**Figure 1:**
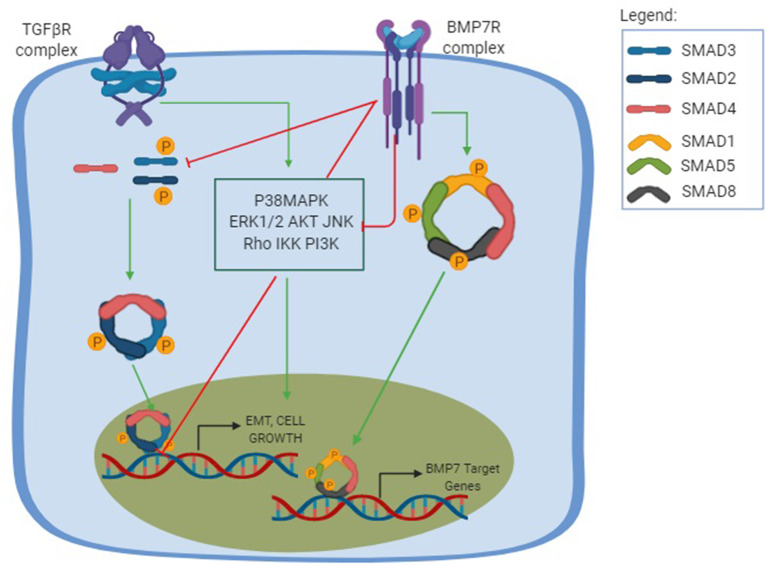
TGF-β and BMP-7 pathways. The TGF-β pathway is shown in its canonical and non-canonical forms. The canonical pathway is Smad-dependent, in which the TGF-β binds to the TGF-βRI and promotes a phosphorylation of TGF-βRII, which will posteriorly recruit downstream signals that results in the phosphorylation of both Smad2 and Smad3 to form a trimer complex with the Smad 4, a common Smad. This results in the trimer translocation into the nucleus and the activation of a number of genes related to cellular functions and profibrotic effect including EMT, cell growth, and apoptosis. The non-canonical pathway, also known as the non-Smad signaling pathways, is mediated by other transducers, such as the p38 mitogen-activated protein kinase (P38MAPK) pathways, that includes each of the extracellular signal regulated kinases (ERKs), AKT, c-Jun amino terminal kinase (JNK), Rho family GTPases, as well as the IkB kinase (IKK), and phosphatidylinositol-3 kinase (PI3K). The non-canonical pathway results in different cellular functions and can amplify the Smad pathway. The BMP-7 pathway is activated after the binding of BMP-7 to the BMP-7R complex and results in the phosphorylation of the Smad1/5/8 to form a complex with the common Smad4 and translocate in the nucleus to activate the opposing function to TGF-β, creating a normal physiological balance between the TGF-β pathway, ultimately leading to an antifibrotic effect, increasing ECM degradation and inducing MET.

BMP-7 was also studied in hepatic fibrosis using mice models where the fibrosis was induced by an injection of carbon tetrachloride (CCL_4_) (106). After the administration of recombinant BMP-7, there was a suppressed progression of liver fibrosis and an improved liver function, partly due to downregulation of type I collagen, α-sma, and TIMP2 and up-regulation of MMP-2 with the suppression of expression of TGF-β (106). Most studies aiming to understand the BMP-7 function were performed on kidney and liver diseases. Meanwhile, rat dermal papilla cells (DPC) treated with BMP-7 demonstrated a counteractive function to the TGF-β profibrotic effect by preventing and reversing the fibrosis (107). We, therefore, strongly relate the opposing relation between BMP-7 and TGF-β in fibrosis and EMT, as schematized in Figure 1. However, whether there is a direct relation between TGF-β or BMP-7 pathways with inflammasome still remains a mystery.

## NLRP3 Inflammasome

In 2002, the research group headed by Jurg Tschopp coined the term “Inflammasome,” defined as a protein complex composed of a nucleotide-binding oligomerization (Nod) receptor, able to activate an inflammatory caspase and, ultimately, lead to IL-1β production (108). Members of the Nod receptor family, which are known as Nod-like receptors (NLRs), have three domains: a carboxy-terminal leucine-rich repeat (LRR), which was first established as the one responsible for sensing the function of the NLRP3 protein (109), but Hafner-Bratkovič and colleagues demonstrated that even with the LRR domain truncation, the inflammasome assembly is still viable, suggesting that its sensor function is probably executed by another domain (110); a central NACHT domain (named after NAIP, CIITA, HET-E, and TP1); and an effector amino-terminal domain, that recruits other proteins with the same domain to exert the signaling function of the oligomer recently formed (111). These domains guarantee the functions of PAMPs and DAMPs recognition, the conformational change, and the formation of oligomers (112).

There are subfamilies of NLRs that differ from each other in the effector domains, with these being the acidic domain (NLRA), BIR domain (NLRB), CARD domain (NLRC), and Pyrin domain (NLRP) (113). Some members of NLRC and NLRP form inflammasome complexes, such as NLRC4, NLRP1, and NLRP3, as well as two other protein receptors, absent in melanoma-2 (Aim-2) and Pyrin (114). An inflammasome complex consisting of NLRC4 does not bind to the ASC adapter protein but instead binds directly to caspase-1 in a card-card interaction since both proteins contain this domain (115). The results from Professor Tschopp's group first brought the notion of a complex containing the cytosolic receptor NALP1 (nowadays renamed as NLRP1) linked to the ASC adapter protein and the inflammatory caspase-1, functioning as a complex that is able to cleave the inactive form of pro IL-1β into its active form (108).

The subfamilies whose amino-terminal domain are Pyrin constitute the major forms of the inflammasome: NLRP1, NLRP3, Aim-2, and Pyrin (116, 117). When activated by an endogenous or exogenous ligand, the proteins bind to each other and interact with the ASC adapter protein, which in turn binds to the inactivated caspase-1. Once cleaved, caspase-1 becomes active and able to cleave protein precursors in aspartate residues, whose main representatives are the cleavage of inactive forms of the pro IL-β and pro IL-18 cytokines, making them active (118). In addition to Pyrin, the ASC protein also contains a CARD domain, responsible for the interaction with the inactive form of caspase-1 (117).

The most studied inflammasome complex to date is the NLRP3 inflammasome. Activation of the NLRP3 inflammasome is divided into canonical and non-canonical. Both pathways present, as a first stage, a response to either TLR activation or some cytokine receptors which are able to promote the transcription of the pro IL-1β and pro IL-18 forms as well as the NLRP3, all via NF-κB, translocation into the nucleus (119). The second step in the canonical pathway is the oligomerization phase of the complex, in which there is recruitment of the adapter protein ASC and pro caspase-1 (119). Subsequently, the complex is activated and performs its function of converting pro IL-1β and pro IL-18 forms into their active forms (120). Besides the cytokine cleavage, the assembly of the NLRP3 inflammasome after its activation and consequent caspase-1 autoclavation, results in the cleavage of a pore forming protein called Gasdermin D (GSMD), resulting in a programmed type of cell death called pyroptosis (121). Pyroptosis is a programmed cell death that is characterized by cell swelling followed by lysis and ending with the release of the intracellular contents.

Once the GSMD is cleaved, it results in the oligomerization of the N terminal domain of the GSDMD which will translocate to the inner cell membrane and bind to the phospholipids (phosphatidylserine and phosphatidylinositol), promoting pore formation and the release of the proinflammatory cytokines IL1β and IL18. As a result, the cell swells and the cytosol content overflows, causing the death of the cell due to a disruption in its osmotic potential. (122). Another phospholipid that GSMD binds to is the cardiolipin, a member of both the bacterial membrane and the mitochondrial outer membrane (122). Rogers and colleagues showed that GSMD-N binding to mitochondrial cardiolipin releases cytochrome C and enhances the apoptosis by the formation of the apoptosome with Apaf-1 and caspase-9 (123, 124).

As mentioned, another result of the pore formation trough GSMD action is the leakage of IL-1β and IL18. Shi and colleagues demonstrated that the release of the IL-1β is increased during pyroptosis. They used *gsmd*^−/−^ Bone Marrow Derived Macrophages (BMDM) and observed a conserved caspase-1 action downstream the NLRP3 activation. Moreover, the secretion of IL-1β in these knockout cells was reduced when compared to wild type ones (121). This IL-1β secretion was later confirmed by Li et al. in an arsenic-induced liver fibrosis model. They showed that arsenate increases GSMD levels and cleavages caspase-1 (125). On the other hand, high expression of miR-379-5p restrained the GSMD expression and the IL-1β expression. Additionally, the EMT appears together with GSMD expression and IL-1β is released by hepatocyte cells (125). However, it is noteworthy that an GSDMD-independent IL1β release does also exist (126, 127).

The non-canonical pathway is a caspase-1 independent pathway and caspase-11 (for mice) or caspase-4/5 -dependent (for humans). Such a pathway, as mentioned before, is initiated after a TLR recognition, specifically, the recognition of a gram-negative bacteria, a group of pathogens that can bind and activate the TLR4-Myd88 and TRIF (Toll-IL-1 receptor homology domain-containing adapter-inducing interferon-β) pathways (128). The consequence of such activation is the translocation of the NF-κB transcription factor into the nucleus, promoting the transcription of NLRP3, pro IL-1β, and pro IL-18, as well the transcription of regulatory factors of interferons 3 and 7 (IRF3 and IRF7). The IRF3 and IRF7 complexes enhance the expression of type I interferons (IFNα/β) which, when binding to its respective receptors, activate the JAK/STAT signaling pathway, leading to the transcription of capsase-11 genes (or caspase-4/5) (120). In different pieces of research it was noted that it is not possible to cleave pro-IL1β and pro-IL18 by caspase-11 but caspase-11 does increase the activity of caspase-1 (129). Moreover, although it is not yet fully known, many data suggest that the pores formed by gasdermin are the predominant point in this non-canonical process (130). In this sense, caspase 11 is capable of initiating the pore forming pyroptosis in a similar way to caspase 1 and thus resulting in the release in the IL1β (131, 132). However, it has been well-noticed that active caspase-11 amplifies the canonical pathway, possibly by processing pannexin 1 and causing potassium efflux (133, 134).

A recent study showed a new integrant of the inflammasome complex, the Nek7 (NIMA-related kinase 7), a known protein involved in mitosis. Schmid-Burgk et al. performed a genomic experiment based on a CRISPR/Cas9 technology that promoted a misfunction of some proteins that were speculated to be involved in the NLRP3 inflammasome activation and regulation. One of these proteins was the Nek7 (135). He et al. demonstrated that Nek7 interacts with NLRP3 but not with any other inflammasome component. They generated Nek7-deficient macrophages and showed no caspase-1 and IL-1β activity (131). More interestingly, the absence of caspase-1 and IL-1β activity happened only in the NLRP3 inflammasome but not in either a NLRC4 or AIM2 inflammasomes activation context (131). Therefore, it was demonstrated that Nek7 interacts with the LRR region of the NLRP3 after ROS inflammasome oligomerization induction (136).

Despite the vast amount of literature concerning the NLRP3 inflammasome, there are still controversial aspects. For example, it was just recently found that the LRR region of the NLRP3 was described as not being required for its activation (110). As the Nek7 binds to the LRR region, this conclusion puts in check the role of this NIMA protein in the inflammasome activation. Another recent description supports a stochastic model for NLRP3 activation, depending on its isoform expressed into the cell, the full-length NLRP3, or the NLRP3 lacking exon 5, that ultimately loses the interaction with Nek7 (137).

### IL-1β and IL-18

IL-1β corresponds to a multifunctional pro-inflammatory cytokine that is secreted by different cell types as a result of the host defense against both infection and injury, whether it is working alone or together with other cytokines (138). It is considered the most popular and the most studied among the family of interleukins (139). IL-1β is released in response to many PAMPs and DAMPs which can activate a variety, or in some cases multiple, PRRs to form inflammasome complexes and is known for its ability to cause fever, hypotension, and the induction of other cytokines such as IL-6 (139). The IL-1β is initially produced in its inactive precursor with 31 KDa in response to different molecular triggers and it is posteriorly cleaved to its 17.3 KDa active form by the pro-inflammatory protease caspase-1 (140), and, once activated, it can be involved in its autocrine and paracrine signaling (138).

There are 10 members of the IL-1 receptors family and of them the IL-1R1 is responsible for binding to IL-1β (141). This recognition happens with the help of a co-receptor IL-1R3, which does not have an IL-1β binding site (141). The IL-1R1 contains a cytosolic functional domain similar to TLRs called Toll-Interleukin receptor (TIR). After the IL-1β binding to IL-1R1, structural changes happen and allow the IL-1R3 to bind and form a trimeric structure (IL-1β, IL-1R1, and IL-1R3) (141). Thereafter, the cytosolic functional domain TIR recruits MyD88 which enables a signaling cascade that promotes NF-κB activation (141).

Inflammasomes and IL-1β are involved in the pathogenesis of many inflammatory disorders, yet the mechanism by which IL-1β is involved in fibrosis is complex. The main hypothesis lies in the relationship between the inflammatory cytokine and TGF-β. According to Luo et al. the short exposure of IL-1β inhibits the action of TGF-β via the NF-κB pathway, and consequently, the Smad pathway, responsible for the transcription of the TGF-β -encoding genes (142). However, this situation is transient due to a change in the NF-κB heterodimer from p65/p50 to p50/p50 at 24 h after exposure to IL-1β (142). The newly formed heterodimer is not able to inactivate Smad and thus, prolonged IL-1β exposure ultimately promotes the expression of TGF-β via Smad (142, 143). Such data represent the first finding regarding the potential of IL-1β in activating TGF-β in vascular muscle cells (144).

Another less discussed cytokine is the IL18 which, like IL1β, is a pro inflammatory cytokine implicated in the pathogenesis of many inflammatory renal diseases (145) and shares a structural homology, produced as a 24-kD inactive precursor lacking a signal peptide (pro-IL-18). This proinflammatory cytokine has proved its relation to EMT in a study performed by Bani-Hani and colleagues, using transgenic mice overexpressing human IL-18-binding protein in HK2 cells, where fibrosis was enhanced by ureteral obstruction, and led to a significant increase in IL-18 production, collagen deposition, α-sma, TGF-β1, and TNF-α production, whereas E-cadherin expression was simultaneously decreased (146). Further analysis revealed that neutralization of IL18 led to reductions in these indicators of obstruction-induced renal fibrosis and EMT, without any alterations in TGF-β1 or TNF-α activity (146).

Another study done by Zhang and colleagues showed that the IL-18 is highly expressed in bleomycin-induced pulmonary fibrosis (147). They used an IL-18 binding protein to block IL-18, and, as result, it prevented the EMT induction by reducing α-sma and reestablishing E-cadherin. The downstream signaling pathway of IL-18 inducing EMT is believed to be the transcriptor factor snail-1, supported by the finding that this factor was upregulated after IL-18 treatment. Also, the snail-1 knockout approach reduces the α-sma and increases E-cadherin with the same IL-18 treatment (147).

## The Role of NLRP3 Activation in the Onset of EMT

**Figure 2:**
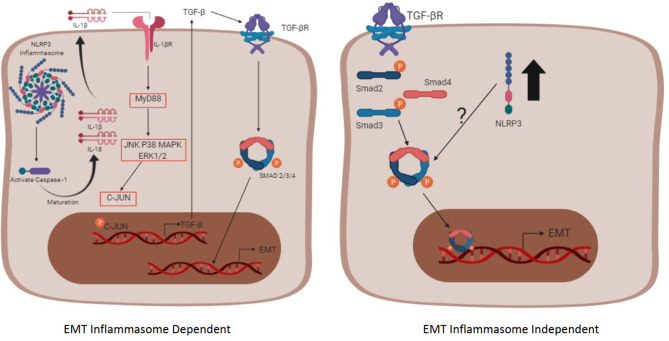
The inflammasome complex -dependent and -independent pathways involved in EMT. The inflammasome complex is activated after two signals (not shown), the first one (priming phase) that promotes the transcription of inflammasome-related proteins (NLRP3, Pro IL-1β, and Pro IL-18) through NF-κB, and the second one (activating phase) that promotes the oligomerization of the complex itself, contributing for the maturation of the cytokines IL-1β and IL-18 through caspase-1. Afterwards, the mature form of IL-1β reaches its receptor in an autocrine, paracrine, or endocrine way and promotes the transcription of the TGF-β gene. The secreted TGF-β binds to its receptor, triggering the pathway dependent of the Smad and promoting the EMT process (Left schematic cell). The pathway independent of the inflammasome oligomerization (right schematic cell) initiates when TGF-β reaches its receptor and, upon the Smad signaling, the nlrp3 expression is enhanced (black arrow). The interaction between the NLRP3 and the Smad3 is not fully understood and requires more studies.

As discussed previously, inflammasome are intracellular complexes formed by receptors of the Nod family, mostly belonging to cells of the innate immune system such as macrophages and dendritic cells. However, few studies show the activation of the complex in epithelium cells of certain tissues, such as the intestine (148), kidneys (149), and spleen (150). In contrast, IL-1β and IL-18 expression has been widely demonstrated to be expressed in tubular epithelial cells (151). Despite the complete relationship between inflammasomes and the onset of fibrosis not being fully known, the inflammasome -dependent and -independent pathways are summarized in Figure 2. The role of NLRP3 inflammasome into the EMT process was questioned by Wang and colleagues in a set of experiments, in which they demonstrated that the EMT process is independent of the NLRP3 inflammasome complex formation, but requires the presence of the NLRP3 protein (152). The levels of *nlrp3* mRNA in mice primary tubular epithelial cells (pTECs) and human proximal tubular cells (HPTC) were found to be increased in response to TGF-β1 (152). Moreover, knockdown of NLRP3 retained an epithelial spindle-like morphology and reversed the mesenchymal characteristics of the cells. Additionally, the TGF-β1-induced MMP-9 and α-sma expression were significantly decreased in NLRP3^−/−^ pTECs. When IL-1β, IL-18, MyD88, and casapse-1 are inhibited, MMP-9 and α-sma expression remains at comparable levels to WT cells (152). These data are also supported by another study demonstrating that the NLRP3 expression, but not the NLRP3 inflammasome complex activation, was required for EMT in colorectal cancer cells, in a model in which cancer cells were transfected with inflammasome-related genes, such as NLRP3. To elucidate the independence of NLRP3 inflammasome complex activation, western blotting analysis was performed and the cleaved form of caspase-1 was not observed after the induction of the EMT process, but only the pro-form of caspase-1 (153).

Recently, some studies have also been elucidating the upstream inflammasome signaling pathways and their role in EMT. One of these studies showed that a medicinal herb called *Huangkui* is able to reduce the mRNA expression of *nlrp3* through diminishing TLR4 and NF-κB protein levels (154). Additionally, another compound, a drug called *paricalcitol*, was suggested to decrease the nuclear translocation of p65 subunit of NF-κB, although the study did not evaluate the inflammasome pathway (155). Years later, another group used the *paricalcitol* in a mesothelial model to investigate the role of NLRP3 in the EMT process. They treated human peritoneal mesothelial cells with TGF-β1 and the addition of *paricalcitol* led to a reduction of *nlrp3* transcription followed by reduced oxidative stress (156). Taken together, these results suggested that *paricalcitol* regulates the EMT process through modulating NLRP3 pathway signaling in an upstream manner, due its action in blocking the NF-κB translocation.

Just like the relationship between fibrosis and NLRP3 inflammasome, the relationship between TGF-β and the NLRP3 inflammasome is not well-understood (152). The understanding that inflammasome complex activation has the function of activating IL-1β and, in turn, IL-1β leads to increased TGF-β expression, brings the idea that the inflammasome complex triggers EMT via TGF-β derived from IL-1β production (157), as shown in Figure 2. It was reported that nanomaterials used in some biosensors, such as nanorods, nanowires, carbonaceous, and rare earth oxides (REOs) are able to activate the NLRP3 inflammasome, and, therefore, trigger EMT in the context of chronic lung fibrosis (158). The relationship between TGF-β and the NLRP3 inflammasome was also demonstrated in an *in vitro* model induced by angiotensin II, a protein able to activate the inflammasome via ROS (157). In this scenario, EMT occurs through the IL-1β signaling since it binds to its receptor and triggers a cascade of signals that promotes the transcription of TGF-β via MyD88 and c-JUN transcription factors. The TGF-β can act in an autocrine and paracrine way by binding to the TGF-βRII that ultimately leads to phosphorylation of Smad2/3 and binding to Smad4, acting as a transcription factor for EMT-related genes (157, 159).

Additionally, there is some evidence showing that IL-1β can enhance TGF-β action and, consequently, lead to EMT in an *in vitro* model using bronchial epithelial cells (160). Dorner and Zuraw treated bronchial epithelial cells with TGF-β alone, or combined with IL-1β, and they observed that when cells were treated with both cytokines, the levels of e-cadherin considerably decreased in comparison with the TGF-β-treated group. Also, the combined treatment raised the levels of tenascin C compared to individual treatment (160). Romero et al. suggests that there is a physical interaction between the NLRP3 and the Smad2/3 transcription factor in the cytosol in order to regulate the expression of genes responsible for EMT (161), which corroborate the results previously described regarding the participation of the NLRP3 inflammasome and the dependence of Smad to lead to EMT (157). It was demonstrated that the involvement of the NLRP3 inflammasome in inducing EMT may be related to the action of TGF-β in renal, pulmonary, and hepatic models of fibrosis (38, 152).

Moreover, Tian et al. demonstrated, in a model of bleomycin-induced pulmonary fibrosis, that the activation of the NLRP3 inflammasome leads to EMT via TGF-β, and once NLRP3 is silenced there are reduced TGF-β levels and, thus, the EMT does not occur (38). The same result was reached by Song et al. in a high glucose induced renal tubular fibrosis (162). Also, a more recent study performed by Li et al. in a silicosis model used the clinically approved drug for treating idiopathic pulmonary fibrosis, *pirefenidone*, and showed it effectively suppressed the EMT process and caused NLRP3 inhibition through phosphorylation of TAK1-MAPK-Snail/NF-κB pathway (40). The relationship between inflammasome and EMT is summarized in Table 1, and, due its controversial consensus, more studies are required in order to clarify results, especially the dependence of the NLRP3 complex formation for the EMT process to occur.

**Table 1:** The relationship between inflammasome and EMT.

**Year**	**Title**	**Authors**	**Main finds**	**Journal**	**References**
2006	Role of caspases on cell death, inflammation, and cell cycle in glycerol-induced acute renal failure	Homsi, E.; Janino, P.; DE Faria, J. B.	Expression of IL-1β and IL-18 in epithelial tubular cells.	Kidney International	(151)
					
2009	TGF-beta1 induced epithelial to mesenchymal transition (EMT) in human bronchial epithelial cells is enhanced by IL-1beta but not abrogated by corticosteroids	Doerner, A. M.; Zuraw, B. L.	IL-1β can enhance TGF-β action and consequently, leads to EMT.	Respiratory Research	(160)
					
2010	Quantitative expression of RIG-like helicase, NOD-like receptor and inflammasome-related mRNAs in humans and mice	Lech, M.; Avila-Ferrufino, A.; Skuginna, V.; Susanti, H. E. et al.	Expression of inflammasome-related genes in epithelium of some organs such as kindney and spleen.	International Immunology	(150)
					
2013	Inflammasome-independent NLRP3 augments TGF-β signaling in kidney epithelium	Wang, W.; Wang, X.; Chun, J.; Vilaysane, A. et al.	EMT is trigger independently of the inflammasome oligomerization, just recquiring the presence of the NLRP3 receptor protein.	Journal of Immunolgy	(152)
					
2014	Renoprotective effect of paricalcitol via a modulation of the TLR4-NF-κB pathway in ischemia/reperfusion-induced acute kidney injury.	Lee, J. W.; Kim, S. C.; Ko, Y. S.; Lee, H. Y. et al.	*Paricalcitol* promotes a decrease on the the nuclear translocation of p65 subunit of NF-κB, which leads to a less expression of inflammasome-related genes.	Biochemical and Biophysical Research Communications	(155)
					
2016	Inflammasome-independent NLRP3 is required for epithelial-mesenchymal transition in colon cancer cells.	Wang, H.; Wang, Y.; Du, Q.; Lu, P. et al.	NLRP3 expression, but not the NLRP3 inflammasome complex activation, was required for EMT in colorectal cancer cells.	Experimental Cell Research	(153)
					
2016	Angiotensin(1-7) attenuated Angiotensin II-induced hepatocyte EMT by inhibiting NOX-derived H2O2-activated NLRP3 inflammasome/IL-1β/Smad circuit	Zhang, L. L.; Huang, S.; Ma, X. X.; Zhang, W. Y. et al.	NLRP3 inflammasome and Smad crosstalk to lead to EMT in renal fibrosis context.	Free Radical Biology and Medicine	(157)
					
2017	Structure Activity Relationships of Engineered Nanomaterials in inducing NLRP3 Inflammasome Activation and Chronic Lung Fibrosis	Wang, X.; Sun, B.; Liu, S.; Xia, T.	Nanomaterials induce NLRP3 activation and enhance, therefore the lung fibrosis.	NanoImpact	(158)
					
2017	NLRP3 participates in the regulation of EMT in bleomycin-induced pulmonary fibrosis.	Tian, R.; Zhu, Y.; Yao, J.; Meng, X. et al.	NLRP3 inflammasome leads to EMT via TGF-β, and once NLRP3 is silenced there are reduced TGF-β levels and thus EMT does not occur.	Experimental Cell Research	(38)
					
2017	Uric acid activates NRLP3 inflammasome in an *in-vivo* model of epithelial to mesenchymal transition in the kidney.	Romero, C. A.; Remor, A.; Latini, A.; De Paul, A. L. et al.	There is a co-localization between NLRP3 and Smad, besides, they showed an expression of all inflammasome proteins expression, suggesting an oligomerization leading to fibrosis.	Journal of Molecular Histology	(161)
					
2018	Knockdown of NLRP3 alleviates high glucose or TGFB1-induced EMT in human renal tubular cells.	Song, S.; Qiu, D.; Luo, F.; Wei, J. et al.	NLRP3 knockdown downregulates the expression of TGF-β1 and blocks the EMT process in high glucose-induced renal fibrosis.	Journal of Molecular Endocrinology	(162)
					
2019	Huangkui capsule alleviates renal tubular epithelial-mesenchymal transition in diabetic nephropathy via inhibiting NLRP3 inflammasome activation and TLR4/NF-κB signaling	Han, W.; Ma, Q.; Liu, Y.; Wu, W. et al.	*Huangkui* reduces the expression of the NLRP3 through the decrease into the TLR4/NF-κB protein levels.	Phytomedicine	(154)
					
2019	Paricalcitol attenuates TGF-β1-induced phenotype transition of human peritoneal mesothelial cells (HPMCs) via modulation of oxidative stress and NLRP3 inflammasome	Ko, J.; Kang, H. J.; Kim, D. A.; Ryu, E. S. et al.	*Paricalcitol* shows a reduction of the *nlrp3* transcription.	The FASEB Journal	(156)

## Conclusion Remarks

The full understanding of the inflammasome complex in the context of the EMT triggering pathway, especially in its relationship with the TGF-β/Smad pathway, remains unclear and requires further study. According to the collected data from the literature, it is suggested that the EMT process might happen in two ways: one is dependent on the inflammasome activation when the IL-1β executes its pro-inflammatory action by up-regulating the TGF-β pathway; another is independent of the inflammasome activation, being necessary only in the presence of the NLRP3 receptor which enhances R-Smads in the TGF-β/Smad pathway. Hopefully, these findings might provide more opportunity for future targeting through blocking fibrosis, or at least retarding it in therapeutic approaches aimed at inflammatory diseases, chronic kidney disease, pulmonary silicosis, or any other disease that is end staged by fibrosis.

## Author Contributions

All authors conceived the ideas present in the paper and wrote the manuscript.

## Conflict of Interest

The authors declare that the research was conducted in the absence of any commercial or financial relationships that could be construed as a potential conflict of interest.
